# Characterization of human aquaporin protein-protein interactions using microscale thermophoresis (MST)

**DOI:** 10.1016/j.xpro.2022.101316

**Published:** 2022-04-15

**Authors:** Tamim Al-Jubair, Jonas Hyld Steffen, Julie Winkel Missel, Philip Kitchen, Mootaz M. Salman, Roslyn M. Bill, Pontus Gourdon, Susanna Törnroth-Horsefield

**Affiliations:** 1 Department of Biochemistry and Structural Biology, Lund University, P.O. Box 124, 221 00 Lund, Sweden; 2 Department of Biomedical Sciences, University of Copenhagen, 2200 Copenhagen, Denmark; 3 College of Health and Life Sciences, Aston University, Aston Triangle, B4 7ET Birmingham, UK; 4 Department of Physiology, Anatomy and Genetics, Kavli Institute for NanoScience Discovery, University of Oxford, Parks Road, OX1 3PT Oxford, UK; 5 Oxford Parkinson’s Disease Centre, University of Oxford, Oxford, UK; 6 Department of Experimental Medical Science, Lund University, P.O. Box 118, 221 00 Lund, Sweden

**Keywords:** Single-molecule Assays, Cell Membrane, Protein Biochemistry

## Abstract

Aquaporin water channels (AQPs) are membrane proteins that maintain cellular water homeostasis. The interactions between human AQPs and other proteins play crucial roles in AQP regulation by both gating and trafficking. Here, we describe a protocol for characterizing the interaction between a human AQP and a soluble interaction partner using microscale thermophoresis (MST). MST has the advantage of low sample consumption and high detergent compatibility enabling AQP protein-protein interaction investigation with a high level of control of components and environment.

For complete details on the use and execution of this protocol, please refer to Kitchen et al. (2020) and Roche et al. (2017).

## Highlights

• Microscale thermophoresis to assay AQP protein-protein interactions
• Quantify interactions with affinities in the sub-nanomolar to millimolar range
• Benefits are low sample consumption and broad compatibility

## Graphical abstract

**Figure:**
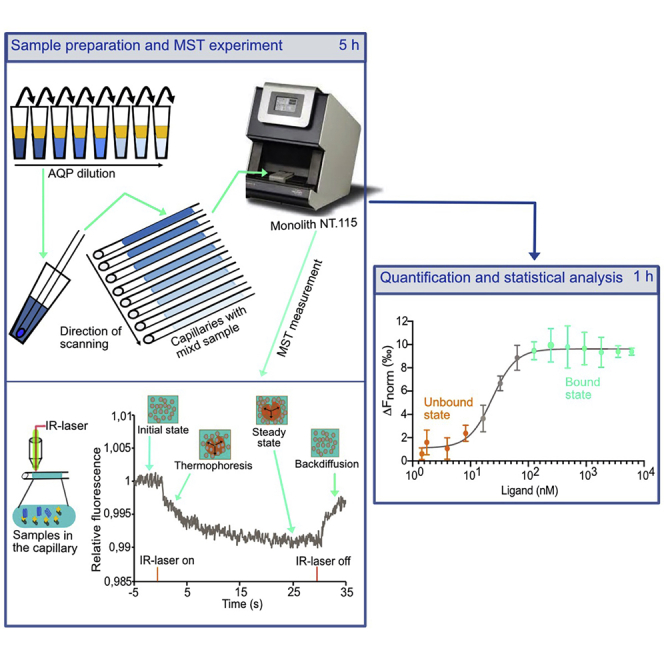

## Teaser

Aquaporin water channels (AQPs) are membrane proteins that maintain cellular water homeostasis. The interactions between human AQPs and other proteins play crucial roles in AQP regulation by both gating and trafficking. Here, we describe a protocol for characterizing the interaction between a human AQP and a soluble interaction partner using microscale thermophoresis (MST). MST has the advantage of low sample consumption and detergent compatibility enabling AQP protein-protein interaction investigation with a high level of control of components and environment.

## Before you begin

Microscale thermophoresis (MST) is based on the principle that the movement of molecules in a thermal gradient depends on their size, charge, and solvation shell. It has the advantage of being fully compatible with a range of buffer conditions, including the presence of detergents, as well as low sample consumption (Jerabek-Willemsen et al., 2011). For these reasons, MST is particularly well suited for studying protein-protein interactions involving membrane proteins. Below we describe a protocol that we routinely use for studying the interaction between full-length human AQPs and a soluble interaction partner.

### Label the soluble interaction partner

**Timing: 1 day**

To follow the formation of the complex, one of the molecules must be fluorescently labeled. Although it is possible to label both the AQP and the interaction partner, we have had the best results when labeling the soluble protein. We typically label our proteins with the cysteine-reactive dye Alexa Fluor 488, however the choice of dye will depend on which amino acids are present in the protein, if any specific locations should be avoided due to potential interference with the interaction and which channels are available on the MST instrument.

1. Label the purified interaction partner with a suitable fluorescent dye, following the manufacturer’s instructions.
2. Incubate the protein at a 2:1 Alexa 488: protein ratio in 20 mM phosphate buffer pH 8 for 3 h at 22°C.
3. Remove any excess unbound dye by passing the sample through a PD-10 desalting column according to the manufacturer’s instructions.
4. Determine the concentration of the labeled protein and concentrate if necessary.

***Note:*** Some dyes, including Alexa Fluor 488, absorb at 280 nm and therefore must be accounted for when determining the concentration.

## Key resources table

**Table:** 

REAGENT or RESOURCE	SOURCE	IDENTIFIER
**Chemicals, peptides, and recombinant proteins**		

AQP interaction partner		
CaCl_2_	Sigma-Aldrich	Cat# 499609-10G
NaCl	Merck	Cat# S9888-5KG
n-Octyl Glucoside Anagrade	Anatrace	Cat# O311HA 25 GM
Tris	Thermo Fisher Scientific	Cat# 17926

**Critical commercial assays**		

Alexa Fluor 488 C5 Maleimide	Invitrogen	Cat# A10254

**Software and algorithms**		

MO.Control	NanoTemper	https://nanotempertech.com/monolith/monolith-nt115/
MO.Affinity Analysis	NanoTemper	https://nanotempertech.com/monolith/monolith- nt115/
Origin	OriginLab	https://www.originlab.com/

**Other**		

PD-10 desalting column	Cytiva	Cat# 17085101
MST Trac Measurement machine	NanoTemper	Model# Monolith NT.115
Monolith NT.115 Premium Capillaries	NanoTemper	Cat# MO-K025

## Materials and equipment

***Alternatives:*** In the key resources table, we have listed the instrument makes and models used in our laboratory. However, these specific models are not crucial for the success of the protocol.

Prepare the solutions accordingly before starting the experiment. Refer to key resources table and materials and equipment sections for a complete list of materials and equipment.

**Table:** MST-buffer (Replace OG with whatever detergent and concentration are most suitable for the protein of interest)

Reagent	Final concentration	Amount
1 M Tris pH 8.0	20 mM	1 mL
5 M NaCl	300 mM	3 mL
OG	1% (w/v)	0.5 g
De-ionized H_2_O		up to 50 mL
**Total**		**50 mL**

Store buffer with the added detergent at 4°C for a maximum of 3 days.

## Step-by-step method details

### Prepare samples

**Timing: 1 h**

1. Determine the required concentration of the fluorescently labeled partner.
  a. Transfer a small amount of the sample to an MST capillary.
  b. Run a capillary scan in the MST instrument (Monolith NT.115).

***Note:*** The signal of the primary capillary scan should be between 200 and 2,000 fluorescence units to ensure a good signal-to-noise ratio without overloading the detector. If necessary, the fluorescence can be adjusted by changing the LED power setting on the MST instrument. Aim for as low concentration as possible that gives an appropriate fluorescence signal. Note that, in the final sample series, the fluorescent partner will be diluted by half, which needs to be considered. If needed, dilute the protein in MST-buffer.

**Figure 1:**
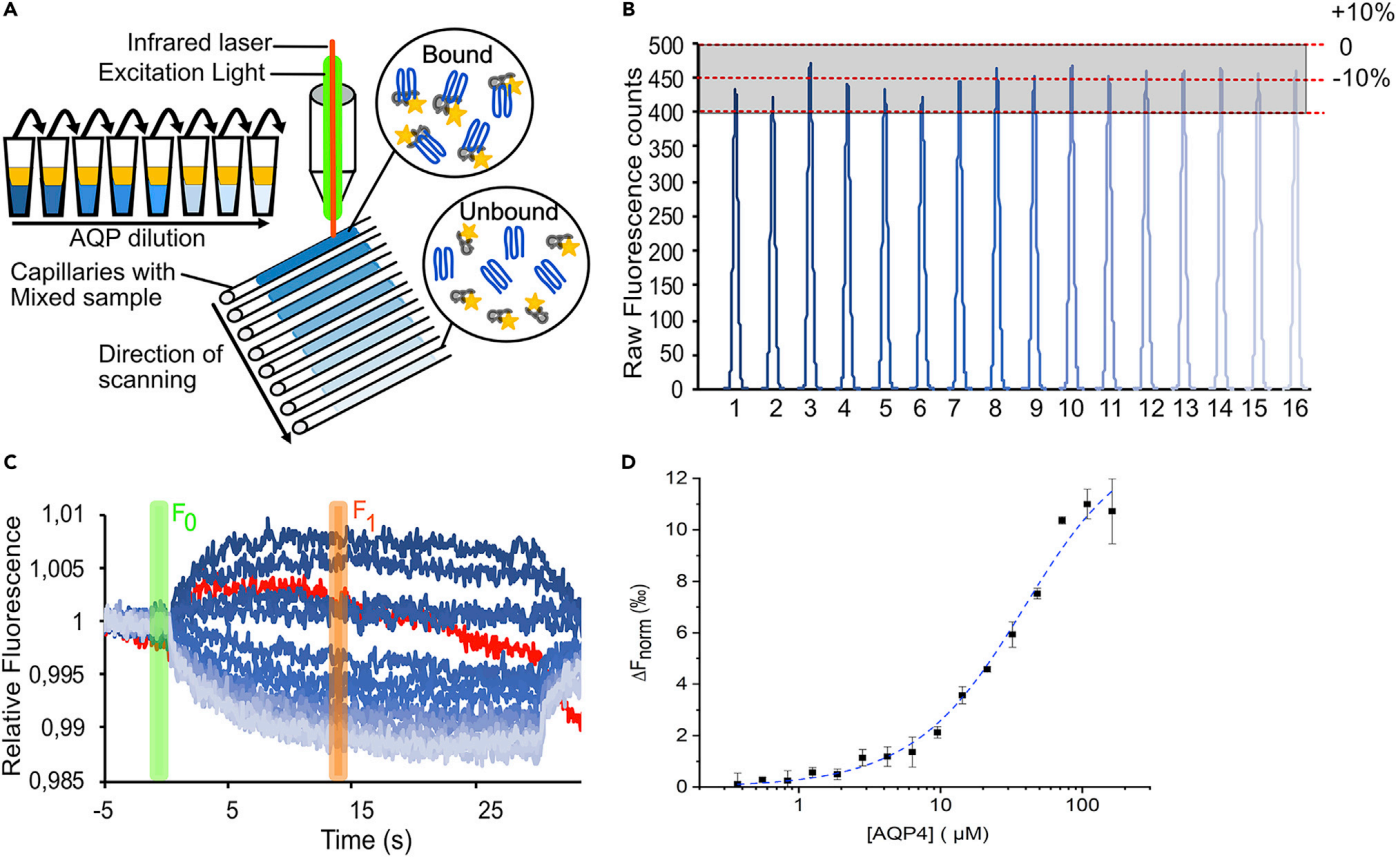
Characterization of AQP protein-protein interactions using MST (A) Dilution series of AQP (blue) mixed with equal amount of fluorescently labeled partner protein (yellow) in small Eppendorf tubes. Mixed samples from the tubes were transferred to capillaries and analyzed by MST. During the experiment, a small part of the sample in each capillary is heated using an infrared laser and the movement of the molecules are followed by fluorescence. (B) A representative capillary scan showing the initial fluorescence in the capillaries before heating. Each peak corresponds to one capillary and are coloured according to the AQP4 concentration, with the highest and lowest concentrations in dark and light blue respectively. The fluorescence level should be the same in all capillaries, with an acceptable deviation of ±10%. (C) MST traces recorded from capillaries loaded with a dilution series of AQP4 mixed with same amount of labeled human calmodulin (CaM). The traces are coloured according to the AQP4 concentration as in (B). A non-well-formed MST trace is shown in red for illustrative purposes. The time points taken as F_0_ (before heating) and F_1_ (after heating) are shown as green and orange boxes respectively. (D) A binding curve generated from MST data, describing the human AQP4-CaM interaction (Kitchen et al., 2020). Curve fitting was done in Origin (OriginLab Corporation) using the full binding equation for a one-to-one model. Error bars represent the standard deviation from three individually prepared sample series.

***Note:*** We typically use a 1:1 dilution series to start with, but it may be advantageous to change to a different dilution ratio to get an optimal binding curve. The AQP starting concentration should be well above the expected dissociation constant (K_d_). If the K_d_ is completely unknown, we suggest using 10 μM as a starting point.

2. Make a dilution series of the purified AQP in MST-buffer in 12–16 200 μL microtubes (Figure 1A). The final volume in each tube should be 5 μL.

3. Add 5 μL of the labeled interaction partner at the concentration determined in step 1.
4. Mix by pipetting up and down a few times.
5. Spin down the samples for 30 s in a tabletop centrifuge.

**CRITICAL:** To minimize fluctuations in fluorescence between samples, add the labeled partner by pipetting gently and mix by pipetting up and down an equal number of times for each tube.

### Run the MST experiment

**Timing: 1 h**

6. Load the samples into MST capillaries by inserting a capillary into the tube and tilting it 45°.

**CRITICAL:** Take care not to introduce any air bubbles as they will disturb the reading of the fluorescence.

7. Load the capillaries into the MST instrument and run a capillary scan to determine the initial fluorescence of the capillaries (Figure 1B).
8. Analyze the fluorescence peaks, making sure that they are uniform in shape and of equal height (see the MST instrument manual for details).

**CRITICAL:** The initial fluorescence as determined in the capillary scan must not differ more than ±10% between capillaries for the MST experiment to be reliable. A random fluctuation of the initial fluorescence is most likely due to pipetting errors whereas a ligand concentration-dependent trend may be caused by the binding event itself, in which case the initial fluorescence values can be used to determine the affinity instead of thermophoretic signal. Alternatively, the concentration-dependent difference can also be due to loss of the fluorescent molecule by sticking to the plastic tubes or capillary walls.

9. Collect MST data and analyze the traces, making sure that they are smooth and do not contain any bumps (this suggests the presence of aggregates) (Figure 1C).

***Note:*** For an initial experiment we recommend collecting MST data at three different MST power settings (e.g., 20%, 40% and 60% MST power) in order to identify the setting that gives an appropriate signal to noise ratio. For collecting final data, we use the lowest MST power setting that gives acceptable MST signal amplitude in the binding curve (ΔF_norm_ ≥ 5).

10. Analyze the data using the MST instrument software (MO Affinity).

***Note:*** The program determines the normalized fluorescence (F_norm_) as the ratio between F_0_ (fluorescence before heating) and F_1_ (fluorescence after heating). If necessary, the time point used for F_1_ can be adjusted to give the best signal to noise ratio.

11. Once the appropriate settings and concentration range have been found, repeat steps 2–9 to generate triplicate data.

**CRITICAL:** Triplicate data should be obtained from three individually prepared dilution series.

## Quantification and statistical analysis

**Timing: 1 h**

1. Use an appropriate curve fitting program and equation to determine the binding affinity by plotting F_norm_ against the ligand concentration (Figure 1D).

***Note:*** If the concentration of the fluorescent partner is not below K_d_, the full binding equation must be used as described below for a one-to-one binding model.

$$y=S1+\left(S2-S1\right)\left(\frac{L_{Free}}{L_{Free}+K_{D}}\right)$$

$$L_{Free}=0.5\;\left(L_{Tot}-P_{Tot}-K_{D}\right)+\;\sqrt{0.25{\left(K_{D}+P_{Tot}-L_{Tot}\right)}^{2}+L_{Tot}K_{D}}$$

S1 and S2 are the signals of the unbound and bound form respectively, L_Free_ and L_Tot_ are the free and total concentration of unlabeled protein (AQP in the experiment described above), P_Tot_ is the total concentration of the labeled protein and K_d_ the dissociation constant.

2. Perform statistical analysis using the three independently prepared dilution series.

## Expected outcomes

Examples of a typical capillary scan, MST traces and a binding curve describing the interaction between human AQP4 and calmodulin (CaM) are shown in (Figure 1). Please note that the shapes of the MST traces may vary depending on the thermophoretic behaviour of the formed complex but should have a smooth and regular trajectory (see MST instrument manual for more details). An example of a poorly formed MST trace (red) is included in Figure 1C for illustration purposes.

## Limitations

The presence of the fluorescent label can have an impact on the binding event. To minimize the risk for this, a fluorescent dye that is not expected to bind to residues in the binding surface should be selected. If necessary, suitable labeling locations can be introduced through genetic engineering, as was done for human CaM (O'Connell et al., 2010). There is also an option for label-free thermophoresis, using the NanoTemper Monolith series, which could be exploited.

If MST is not available, there are several alternative techniques for studying protein-protein-interactions, most notably isothermal titration calorimetry (ITC) and surface plasmon resonance (SPR). However, these techniques come with their own limitations that must be taken into consideration, for example the disturbance from detergent micelles in ITC and the immobilization of molecules in SPR. In our laboratory, we have successfully used fluorescence anisotropy (Kreida et al., 2018) and fluorescence spectroscopy (Roche et al., 2019) as an alternative to MST for characterizing interactions between AQPs and other proteins.

## Troubleshooting

### Problem 1

Air bubbles are introduced during sample transfer to capillaries (step 6).

### Potential solution

Empty the capillary back into the same tube and repeat the transfer procedure. If the bubbles persist, making sure that they are not in the middle of the capillary is usually sufficient to allow for an appropriate fluorescence reading.

### Problem 2

There is a random fluctuation in the initial fluorescence of the samples during the capillary scan (steps 7–8).

### Potential solution

Redo the sample preparation in steps 2–5, taking extra care when pipetting the fluorescently labeled partner.

### Problem 3

The capillary scan indicates ligand concentration-dependent fluctuation of the initial fluorescence, but it is not known if this is due to ligand binding or sample issues (steps 7–8).

### Potential solution

Perform a standard denaturation test as follows: Prepare samples in the same manner as in the MST experiment at two concentrations of unlabeled protein for which there is a maximum difference in initial fluorescence. Typically, this would correspond to high (saturating) and low (non-binding) concentrations of the unlabeled protein. Centrifuge the samples at 13,000 × *g* for 10 min to remove any aggregates. Mix the samples with an equal volume of denaturing solution (4%(w/v) SDS, 40 mM DTT) and heat at 95°C for 5 min. Centrifuge briefly and transfer the sample to capillaries. Measure the fluorescence of the capillaries using the same settings as in the MST experiment. If the fluorescence of the two samples is equal after denaturation, this suggests that the concentration-dependent variation was induced by binding rather than surface adsorption or aggregation. In this case, use the fluorescence values instead of the MST signal to create a binding curve.

### Problem 4

There is a concentration-dependent variation in initial fluorescence that is not due to binding (steps 7–8).

### Potential solution

Mix the labeled binding partner with a 10-20-fold molar excess of the same protein in an unlabeled state. Use this mixture as the “labeled protein sample” when preparing the MST samples. The unlabeled protein replaces the labeled protein in surface adsorption processes, resulting in a reduced loss of fluorescent molecules. Please note that the total concentration of the interaction partner (labeled + unlabeled) must be used in the binding equation when calculating K_d_.

### Problem 5

The MST-trajectories are bumpy, indicating the presence of aggregates (step 9).

### Potential solution

Centrifuge the samples at 13,000 × *g* for 5 min at 4°C before transferring them to the capillaries.

## Resource availability

### Lead contact

Further information and requests for resources and reagents should be directed to and will be fulfilled by the lead contact, Prof. Susanna Törnroth-Horsefield Email: Susanna.Horsefield@biochemistry.lu.se.

### Materials availability

All unique/stable reagents generated in this study are available from the lead contact with a completed Materials Transfer Agreement.

## Data Availability

This study did not generate/analyze datasets and codes.
